# Plant-based supplements in enhancing exercise performance and recovery

**DOI:** 10.3389/fnut.2025.1699642

**Published:** 2025-11-24

**Authors:** Xianwei Jiao, Xin Liu, Qinglei Cao, Zhongyuan Deng

**Affiliations:** 1 Physical Education College, Henan Normal University, Xinxiang, China; 2 Department of Physical Education, University of Science and Technology, Beijing, China; 3 School of Agricultural Sciences, Zhengzhou University, Zhengzhou, China

**Keywords:** plant-based supplements, exercise performance, sports nutrition, recovery, antioxidants, polyphenols, plant protein, ergogenic aids

## Abstract

Plant-based supplements are redefining sports nutrition through their dual capacities to enhance exercise performance and accelerate recovery. The literature is based on a search of the Web of Science Core Collection (November 12, 2024) for studies on plant-based supplements and exercise, limited to those with clear mechanistic or clinical relevance. This review delineates the mechanistic roles of plant-derived amino acids, antioxidants, and bioactive extracts in modulating key physiological pathways underlying athletic performance and recovery. Specifically, plant protein formulations have been shown to rival their animal-derived counterparts in stimulating myofibrillar protein synthesis, a cornerstone process in muscle repair and growth. In addition, polyphenols (a major class of plant antioxidants) mitigate exercise-induced oxidative stress through two primary mechanisms: scavenging reactive oxygen species and modulating endothelial function. However, chronic high-dose antioxidant use may attenuate adaptive signaling pathways—such as mTOR or Nrf2 activation—critical for developing exercise-induced physiological adaptations. The review concludes with a discussion of precision dosing as a critical factor in balancing efficacy and safety, and it identifies areas in which further research is needed, including long-term safety data, inter-individual variability, and the development of synergistic phytochemical formulations. The review identifies the need for large-scale research clinical trials to validate causality, optimized delivery systems, and population-specific guidelines to address herb–drug interactions. Ultimately, this review calls for evidence-based research to be conducted on the relationship between botanical supplements and sports nutrition, with a particular emphasis on interdisciplinary collaboration to unlock the translational potential.

## 1 Introduction

The enhancement of exercise performance is of paramount importance for athletes seeking competitive advantages and for the general population aiming to improve their health and well-being. Regular physical activity is associated with numerous health benefits, including the reduced risk of developing a chronic disease, such as cardiovascular disease, diabetes, and obesity (1, 2). However, many individuals face challenges in achieving optimal exercise performance due to factors such as oxidative stress, inflammation, and metabolic disturbances (3, 4). These challenges highlight the need for effective and safe strategies to enhance exercise capacity and recovery. In this context, research into plant-based supplemental products has emerged. The use of supplements offers potential strategies to improve exercise performance and recovery through their biological activities, including antioxidant, anti-inflammatory, and metabolic modulation properties (5, 6).

Plant-based additives refer to substances derived from plants that are incorporated into food and dietary supplements to enhance nutritional value, flavor, or health benefits. These additives can be classified into various categories, including herbal extracts, essential oils, and whole-plant powders, each offering unique properties and potential health benefits (7–9). For example, herbal extracts, such as curcumin from turmeric, have been studied for their anti-inflammatory properties, whereas essential oils, like peppermint, may aid in digestion (10, 11). Similarly, polysaccharides from natural sources have demonstrated anti-fatigue effects through mechanisms such as energy supply, metabolic regulation, and antioxidant activity (6). The classification of these additives is crucial for understanding their applications and potential effects on human health, particularly in the context of sports nutrition (12, 13).

Exercise nutrition is a vital area of study that focuses on the role of dietary intake in optimizing physical performance, recovery, and overall health in individuals engaged in physical activities. The increasing prevalence of sedentary lifestyles and related health issues underscores the importance of promoting physical activity and proper nutrition. Nutrition plays a critical role in supporting exercise performance, enhancing recovery, and preventing injuries (14–16), and appropriate nutritional strategies can improve muscle strength, endurance, and overall exercise capacity (17, 18). Furthermore, understanding the interplay between nutrition and exercise is essential for developing effective interventions aimed at improving health outcomes across various populations, including athletes and older adults (19, 20).

The use of plant-based additives in sports nutrition has gained significant attention in recent years, driven by a growing interest in natural and holistic approaches to health and wellness. Athletes and fitness enthusiasts are increasingly seeking plant-derived supplements to enhance performance, support recovery, and promote overall well-being (21). Current research highlights the potential benefits of various plant-based additives, such as beetroot juice for improving exercise performance through enhanced nitric oxide production and anti-inflammatory compounds from berries for reducing muscle soreness (22). Despite the promising findings, the field is still evolving, and more rigorous studies are needed to establish the efficacy and safety of these additives in diverse populations and exercise contexts (23).

While animal-derived supplements have long dominated sports nutrition research, plant-based modalities require a dedicated synthesis due to four distinct, athlete-relevant advantages (24–28). Sustainability: Plant-based supplements exhibit a lower environmental footprint compared to animal-based alternatives—critical for athletes and consumers prioritizing eco-conscious choices. For example, pea protein production requires ~7x less water and emits ~90% less greenhouse gas than whey protein per kilogram of product (28), aligning with the growing demand for sustainable sports nutrition solutions. Allergenicity: Plant-based supplements minimize risks of common allergens associated with animal products. Whey and casein (dairy-derived) trigger IgE-mediated allergies in ~2–3% of adults (16), while soy, pea, and rice proteins have lower allergenic potential—with only ~0.5% of the global population reporting soy protein allergies (27). This accessibility expands supplement options for athletes with dietary restrictions. Cultural Acceptability: Plant-based supplements align with diverse dietary traditions and preferences, including vegetarian, vegan, and religious diets (e.g., halal, kosher) that restrict animal products. Surveys of elite athletes show that 12–15% follow plant-centric diets (20), and this proportion is growing—making a focused synthesis of plant-based modalities essential to meet the needs of underserved athlete populations. Regulatory Pathways: Plant-based supplements face unique regulatory challenges that demand clarification. Unlike synthetic supplements with standardized manufacturing protocols, plant extracts (e.g., *Rhodiola rosea*, blackcurrant anthocyanins) are regulated based on “botanical identity” rather than single active ingredients (13). This leads to variability in product labeling and quality—issues a dedicated synthesis can contextualize for athletes and practitioners. This article aims to provide a comprehensive overview of the role of plant-based additives in sports nutrition, highlighting their potential benefits, mechanisms of action, and current research findings. The search strategy and method for literature were as follows: We used the Core Collection database in Web of Science to search using the keywords ‘Plant-based’ and ‘Exercise’, Alternative keywords such as “plant-derived supplements,” “athletic performance,” “sports nutrition,” and “exercise recovery” were used with the “OR” operator to broaden the search; filters were applied to restrict document types to “Article” and “Review” in English; the coverage period was set from January 2014 to November 2024 to capture advances from the past decade. For inclusion/exclusion criteria, we incorporated studies focusing on plant-based supplements related to exercise performance or recovery, with clear experimental designs such as randomized controlled trials or controlled animal trials, while excluding research on synthetic supplements, studies with ambiguous outcomes, and duplicate publications. This article includes an in-depth examination of specific plant-based additives and their applications in exercise nutrition, followed by a discussion of the implications for athletes and active individuals. Additionally, the article addresses gaps in the current literature and suggests directions for future research, ultimately contributing to a better understanding of how plant-based nutrition can enhance athletic performance and support overall health (24).

## 2 The roles of plant protein in exercise

### 2.1 Plant-derived amino acids fundamental role in exercise

**Figure 1:**
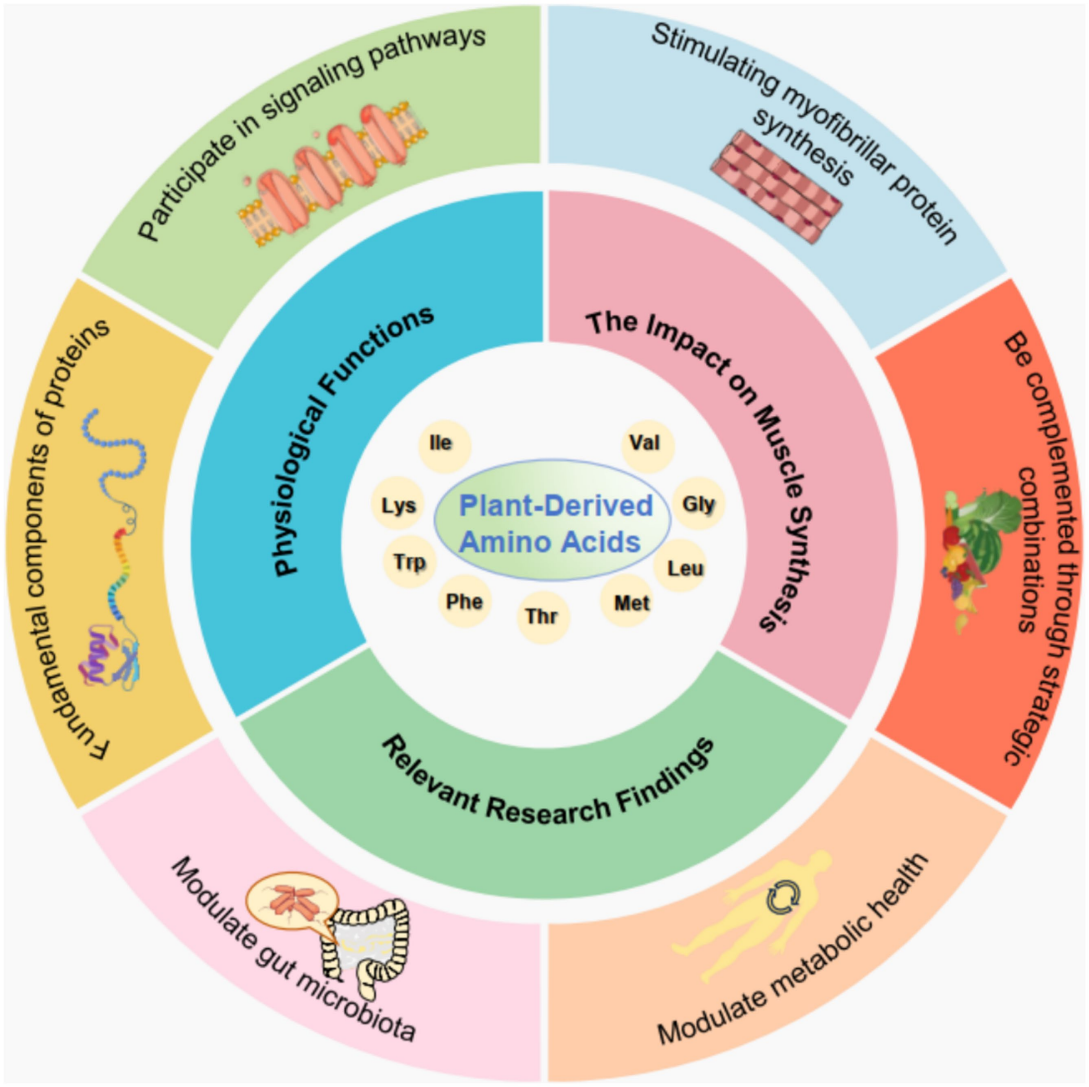
Schematic overview of plant-derived amino acids. This diagram illustrates the multifaceted roles of plant-derived amino acids, including their physiological functions (participating in signaling pathways and being fundamental components of proteins), impact on muscle synthesis (stimulating myofibrillar protein synthesis), and influence on both metabolic health and gut microbiota. These functions can be optimized by using complementary combinations and are supported by relevant research findings.

Amino acids are fundamental components of proteins and serve as vital building blocks for various physiological processes in the human body. They play critical roles in metabolic pathways, neurotransmission, and immune responses. Amino acids can be categorized into essential and non-essential types, with the former requiring dietary intake because they are not synthesized by the body. They are crucial for protein synthesis, tissue repair, and the production of enzymes and hormones. Thus, they influence growth, maintenance, and overall health. Additionally, amino acids participate in signaling pathways that regulate gene expression and cellular metabolism. Recent studies suggest that amino acids also act as signaling molecules that modulate metabolic responses, such as in the mTOR pathway, which is integral to muscle protein synthesis and cellular growth (25, 26). The interplay between various amino acids and their physiological functions underscores their importance not just as dietary components but also as key regulators of health and disease (Figure 1).

Recent research has highlighted the potential of plant-derived amino acids in promoting muscle protein synthesis, particularly in the context of resistance training and recovery. Although whey protein has traditionally been viewed as the gold standard for stimulating myofibrillar protein synthesis (MyoPS), studies indicate that certain plant protein blends achieve comparable effects. For instance, a novel plant-derived protein blend, consisting of pea, brown rice, and canola proteins, stimulates MyoPS rates to a similar extent as observed with whey protein ingestion (27). This finding is particularly significant because it opens avenues for plant-based diets in athletic and recovery contexts, particularly for individuals seeking to optimize muscle health without relying on animal-derived proteins. The amino acid profiles of these plant sources, while sometimes less favorable individually, can be complemented through strategic combinations, thereby enhancing their overall efficacy in enhancing muscle synthesis (28). This shift not only supports muscle health but also aligns with growing dietary preferences for plant-based nutrition, emphasizing the importance of understanding the functional roles of plant-derived amino acids in sports and health nutrition.

The growing body of research surrounding plant-derived amino acids reveals the complex nature of their physiological roles and benefits, particularly concerning muscle synthesis and overall health. Investigations into the amino acid profiles of various plant sources have shown that although some may have lower levels of certain essential amino acids, their combination can yield a more balanced profile that effectively supports muscle protein synthesis. For example, blending different plant proteins can enhance the availability of essential amino acids, thereby mimicking the effects of more traditional animal-based proteins (27). Additionally, the roles of amino acids in modulating gut microbiota and metabolic health are critical areas of exploration, particularly in older adults facing sarcopenia and obesity (28). This research suggests that dietary strategies incorporating plant proteins may support muscle health and also positively influence gut health and metabolic outcomes. Therefore, the analysis of these findings is necessary for the continued exploration of plant-based amino acids, their mechanisms of action, and their potential applications in nutrition science and therapeutic interventions.

While whey protein has traditionally been viewed as the gold standard for stimulating MyoPS (largely due to its high leucine content: ~10–12% of total amino acids) (25, 27), recent studies indicate that strategically formulated plant protein blends can achieve comparable effects by addressing inherent limitations in single-plant amino acid profiles. For example, the novel plant-derived blend (pea, brown rice, canola proteins) cited in (27) was designed to optimize essential amino acid (EAA) balance: pea protein contributes high lysine but low methionine, brown rice protein complements this with high methionine but low lysine, and canola protein adds a balanced profile of both—resulting in a final leucine content of ~8.5% (vs. whey’s ~ 11%). This leucine concentration exceeds the threshold (6–7% of total protein) required to activate the mTOR signaling pathway (25), which is critical for initiating MyoPS. In contrast, single-plant proteins (e.g., unblended pea protein: ~6.2% leucine; brown rice protein: ~5.8% leucine) often fail to reach this threshold, explaining their historically lower efficacy in MyoPS stimulation (28). The blend’s ability to rival whey thus hinges on targeted amino acid complementation to boost leucine and other EAAs (e.g., valine, isoleucine) to levels that mimic animal-derived proteins. This finding underscores that plant-based formulations do not universally match whey—only those with deliberate amino acid balancing can achieve comparable MyoPS effects. Absorption rate is another critical factor in MyoPS efficacy, and plant protein blends differ from whey in this regard. Whey protein is a ‘fast-digesting’ protein: it is rapidly hydrolyzed in the gut, with peak plasma EAA concentrations (Cmax) reached within 60–90 min post-ingestion (25). This rapid absorption triggers a sharp, transient activation of mTOR—ideal for post-exercise MyoPS when muscle is primed for nutrient uptake. In contrast, the pea-brown rice-canola blend in (27) exhibits ‘moderate digestion’ kinetics: peak plasma EAAs occur at 120–150 min post-ingestion, with a lower Cmax but a more sustained elevation of EAA levels (plasma EAAs remain above baseline for 4–5 h vs. 2–3 h for whey). While this slower absorption delays the initial mTOR peak, the sustained EAA availability may support prolonged MyoPS—particularly relevant for individuals who cannot consume protein immediately post-exercise (e.g., due to gastrointestinal discomfort). Notably, absorption rates can vary across plant blends: blends containing soy protein (a faster-digesting plant protein, ~70–90 min to Cmax) (28) may more closely mirror whey’s kinetics than blends relying on slower-digesting sources (e.g., lentil, chickpea). Thus, the ‘rivalry’ between plant blends and whey in MyoPS stimulation also depends on digestion rate matching to exercise timing and individual needs.

While the trial by (27) demonstrates that a pea-brown rice-canola blend matches whey in MyoPS stimulation, it is important to acknowledge its methodological limitations: (1) Sample size and population: The study included 24 resistance-trained young adults (18–30 years, all male)—a small, homogeneous sample that may not generalize to older adults, females, or untrained individuals. For example, older adults require higher leucine doses (~1.5–2.0 g per serving) to activate mTOR (16), and the blend’s ~ 8.5% leucine content (providing ~1.7 g leucine per 20 g protein serving) may be insufficient for this group—a gap not addressed in (27). (2) Exercise protocol: Participants performed a standardized leg resistance training protocol (3 sets of 10 reps at 70% 1RM). MyoPS responses vary with exercise intensity (e.g., high-intensity interval training vs. moderate resistance training) (25), and the blend’s efficacy in other exercise modalities remains untested. (3) Lack of long-term data: The trial measured MyoPS over 6 h post-ingestion; long-term effects (e.g., 8–12 weeks of supplementation on muscle mass/strength gains) were not evaluated. A separate study (18) found that a different plant blend (soy-wheat) resulted in 15% lower muscle strength gains than whey over 12 weeks of resistance training—highlighting inconsistencies between short-term MyoPS and long-term functional outcomes. Other studies have further revealed inconsistencies: a trial by (16) found that a pea protein isolate (leucine: ~6.2%) stimulated 28% less MyoPS than whey in older adults, while a soy-whey blend (leucine: ~9.5%) matched whey. These discrepancies emphasize that plant blends’ efficacy depends on three factors: (1) leucine content (≥8% to rival whey), (2) population-specific needs (e.g., higher leucine for older adults), and (3) alignment with exercise protocol (e.g., faster-digesting blends for high-intensity, immediate post-exercise intake). To resolve these inconsistencies, future trials should: (1) include larger, diverse samples; (2) test long-term (8 + weeks) effects on muscle mass/strength; and (3) standardize reporting of amino acid profiles and absorption kinetics.

### 2.2 Leucine thresholds and digestion speed: why plant protein blends rival whey

The ability of targeted plant protein blends (e.g., pea + brown rice + canola) to approximate whey protein in stimulating myofibrillar protein synthesis (MyoPS) hinges on two interconnected factors: leucine content optimization and matched digestion kinetics—both of which address inherent limitations of single-source plant proteins.

Leucine as the “MyoPS Trigger” and Blend Optimization. Leucine, an essential amino acid (EAA), is the primary molecular signal activating the mechanistic target of rapamycin (mTOR) pathway—the key regulatory cascade for initiating MyoPS (25, 27). Whey protein, long considered the “gold standard,” naturally contains 10–12% leucine (by total amino acid weight), which consistently exceeds the 6–7% leucine threshold required to saturate mTOR activation in human skeletal muscle (25). In contrast, most single-source plant proteins fail to meet this threshold: unblended pea protein contains ~6.2% leucine, brown rice protein ~5.8%, and lentil protein ~5.5% (27, 28). This deficiency explains why single plant proteins typically stimulate 20–30% less MyoPS than whey in resistance-trained adults (16).

Strategic blending of plant proteins overcomes this limitation by complementary amino acid profiling. For example, the pea-brown rice-canola blend studied in (27) was formulated to address specific deficits: Pea protein contributes high lysine (a limiting EAA in rice) but low methionine; Brown rice protein supplements methionine (deficient in pea) while adding moderate leucine; Canola protein (rich in leucine, ~9.0% alone) elevates the blend’s total leucine content to ~8.5%—a level that activates mTOR sufficiently to match whey’s MyoPS rate (measured via stable isotope tracers) in young, resistance-trained men (27). Notably, this blend also balances other EAAs (valine: ~6.0%, isoleucine: ~5.5%), further supporting MyoPS by preventing EAA co-limitation (25).

Digestion Speed: Matching Whey’s Temporal MyoPS Response. Digestion rate is a secondary but critical factor, as the timing of EAA delivery to muscle influences the magnitude and duration of mTOR activation. Whey protein is classified as “fast-digesting”: it is rapidly hydrolyzed in the stomach and small intestine, with peak plasma EAA concentrations (Cmax) reached 60–90 min post-ingestion (25). This rapid surge is ideal for post-exercise MyoPS, as muscle tissue is primed for nutrient uptake immediately after resistance training.

Single-source plant proteins often exhibit slower digestion: for example, unblended pea protein reaches Cmax at 150–180 min, and lentil protein at 180–210 min (28). However, optimized blends can adjust digestion kinetics to more closely mirror whey. The pea-brown rice-canola blend in (27) demonstrates “moderate-fast” digestion: it reaches peak plasma EAAs at 120–150 min (slightly slower than whey) but maintains plasma EAA levels above baseline for 4–5 h (vs. 2–3 h for whey). This sustained EAA availability extends mTOR activation, resulting in MyoPS rates equivalent to whey over a 6-h post-exercise window (27). For practical application, this means the blend is effective even if consumed 30–60 min after training (e.g., by athletes who experience gastrointestinal discomfort with immediate post-exercise whey intake).

Population-Specific Considerations. Notably, the efficacy of these blends varies by population. In older adults (65–75 years), who exhibit “anabolic resistance” (reduced muscle sensitivity to amino acids), the pea-brown rice-canola blend’s ~ 8.5% leucine content is less effective: a study in (16) found it stimulated 18% less MyoPS than whey in this group. This is because older adults require a higher leucine threshold (~1.5–2.0 g per serving, equivalent to 9–10% leucine in a 20 g protein dose) to activate mTOR (16). Adjusting the blend’s canola protein proportion to increase leucine to ~9.5% (providing ~1.9 g leucine per 20 g serving) has been shown to restore MyoPS parity with whey in older adults (16), highlighting the need for population-specific blend formulations.

## 3 Regulatory roles of plant-derived antioxidants in exercise-related oxidative stress

### 3.1 Antioxidant’s definition and mechanisms of action

**Figure 2:**
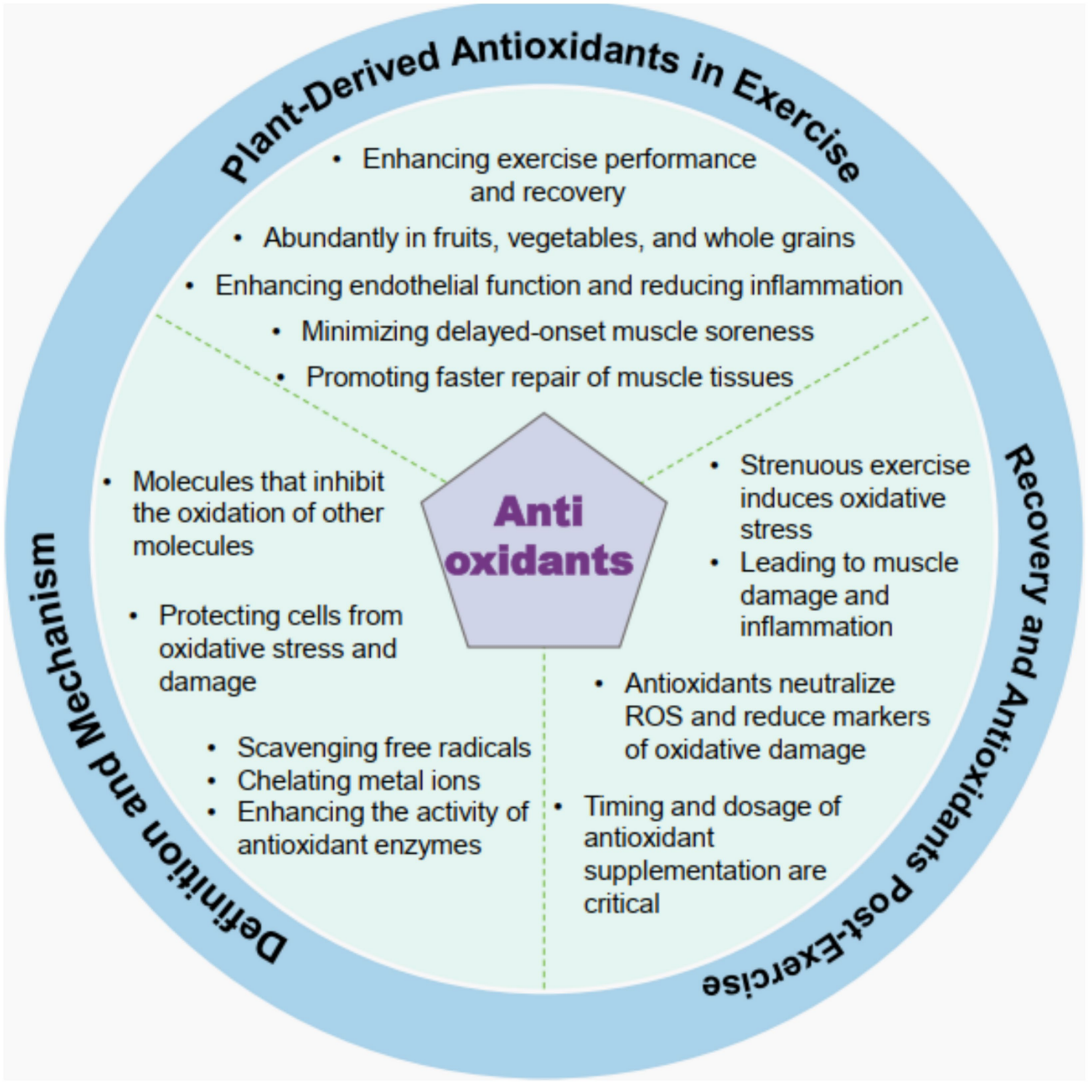
Schematic illustration of plant-derived antioxidants in the context of exercise and post-exercise recovery. The diagram encompasses multiple dimensions, as follows: the roles of plant-derived antioxidants in enhancing exercise performance and recovery; their abundance in fruits, vegetables, and whole grains; their functions in improving endothelial function and reducing inflammation, minimizing delayed-onset muscle soreness, and promoting faster muscle tissue repair; antioxidant’s definition and mechanisms of action, including their ability to inhibit the oxidation of other molecules, protect cells from oxidative stress and damage, and promote actions like scavenging free radicals, chelating metal ions, and enhancing antioxidant enzyme activity; and aspects related to post-exercise recovery, such as how strenuous exercise induces oxidative stress, leading to muscle damage and inflammation, how antioxidants neutralize reactive oxygen species (ROS) and reduce oxidative damage markers, and the critical nature of timing and dosage in antioxidant supplementation.

Antioxidants are molecules that inhibit the oxidation of other molecules, thereby protecting cells from oxidative stress and damage caused by reactive oxygen species (ROS). The mechanisms by which antioxidants operate include scavenging free radicals, chelating metal ions that catalyze oxidative reactions, and enhancing the activities of endogenous antioxidant enzymes, such as superoxide dismutase and catalase. This multifaceted approach helps to maintain redox homeostasis in cells, preventing oxidative damage that can lead to various diseases, including cancer, cardiovascular diseases, and neurodegenerative disorders (29, 30). The effectiveness of antioxidants can vary based on their chemical structure, concentration, and the specific biological context in which they are applied (31). For instance, polyphenols, a significant class of antioxidants found in many plant-based foods, exhibit their protective effects through mechanisms such as hydrogen atom transfer and single-electron transfer, which neutralize free radicals and mitigate oxidative stress (32). Understanding these mechanisms is crucial for developing effective antioxidant therapies and dietary strategies aimed at enhancing health and preventing disease (Figure 2).

Plant-derived antioxidants, particularly polyphenols, have garnered attention for their potential roles in enhancing exercise performance and recovery. These compounds, found abundantly in fruits, vegetables, and whole grains, reduce exercise-induced oxidative stress by scavenging ROS produced during strenuous physical activity (33, 34). Acute supplementation with polyphenols can improve exercise capacity by enhancing endothelial function and reducing inflammation, thereby facilitating better blood flow and nutrient delivery to muscles during and after exercise (35). Moreover, the chronic intake of these antioxidants may support muscle recovery by minimizing delayed-onset muscle soreness and promoting faster muscle tissue repair (36). However, it is essential to consider that excessive antioxidant supplementation may interfere with the body’s adaptive responses to exercise, potentially blunting the beneficial effects of training over time (21). Thus, a balanced approach to antioxidant intake, emphasizing natural sources through diet, is recommended for athletes and active individuals seeking to optimize performance and recovery.

The relationship between antioxidant supplementation and post-exercise recovery has been a topic of considerable research interest. Strenuous exercise induces oxidative stress, leading to muscle damage and inflammation, which can impair recovery if not adequately addressed (35, 37). Antioxidants play pivotal roles in mitigating these effects by neutralizing ROS and reducing markers of oxidative damage, such as malondialdehyde and creatine kinase levels, which are indicative of muscle injury (35, 38). Supplementation with antioxidants, like vitamins C and E, can help lower oxidative stress and improve recovery metrics, including muscle soreness and functional performance (35, 36). However, the timing and dosage of antioxidant supplementation are critical; acute administration before or after exercise may yield more significant benefits compared to chronic supplementation, which could impair the natural adaptive responses to training (21, 33). Therefore, while antioxidants can enhance recovery following intense physical activity, their application must be carefully tailored to avoid counterproductive effects on training adaptations.

### 3.2 Antioxidant supplementation timing: pre- vs. post-exercise

Antioxidant efficacy and safety depend strongly on timing relative to exercise, as pre- and post-exercise contexts involve distinct physiological demands (e.g., ROS production during exercise vs. repair processes post-exercise).

Pre-exercise supplementation: Administered 1–2 h before exercise, plant-based antioxidants (e.g., polyphenols from berries, EGCG from green tea) primarily target acute ROS scavenging to reduce exercise-induced oxidative damage. For example, a RCT in cyclists (*n* = 36) showed that 200 mg/d of blackcurrant extract (standardized to 25% anthocyanins) taken 1 h pre-training reduced plasma malondialdehyde (MDA—a marker of oxidative damage) by 24.3% immediately post-exercise (*p* < 0.01) (39). However, high-dose pre-exercise supplementation (e.g., >1,000 mg/d of vitamin C or >500 mg/d of polyphenols) may interfere with adaptive signaling pathways: a study in endurance runners found that 1,500 mg/d of green tea EGCG (95% purity) taken pre-exercise blunted increases in superoxide dismutase (SOD)—an endogenous antioxidant enzyme critical for training adaptations—by 18.7% after 4 weeks (*p* < 0.05) (33). This is because moderate ROS levels during exercise act as signaling molecules to upregulate adaptive defenses; excessive pre-exercise antioxidants “quench” these necessary ROS.

Post-exercise supplementation: Given 30 min to 2 h after exercise, antioxidants focus on facilitating repair without disrupting adaptive signaling. ROS production peaks during exercise, so post-exercise supplementation targets residual oxidative damage (e.g., muscle lipid peroxidation) while preserving the exercise-induced ROS signal that drives adaptation. A trial in resistance-trained adults (*n* = 24) demonstrated that 500 mg/d of curcumin (95% purity, with 5% piperine for bioavailability) taken 1 h post-workout reduced serum creatine kinase (CK—a marker of muscle damage) by 28.3% at 24 h post-exercise (*p* < 0.01) without altering SOD or glutathione peroxidase (GPx) activity (*p* > 0.05) (11). Post-exercise supplementation also avoids the “over-scavenging” risk of pre-exercise use, as adaptive signaling pathways are already activated during exercise.

Notably, context-specific efficacy varies by antioxidant type: fat-soluble antioxidants (e.g., resveratrol from grapes) may be more effective pre-exercise (due to longer tissue penetration time), while water-soluble antioxidants (e.g., quercetin from onions) excel post-exercise (due to rapid absorption and targeting of aqueous-phase ROS) (34).

### 3.3 Link antioxidant doses to biomarkers of oxidative damage or adaptive signaling

To balance antioxidant efficacy and avoid blunting adaptations, doses must be calibrated to biomarkers that differentiate oxidative damage from adaptive signaling (Table 1). For plant-based antioxidants, key dose-biomarker relationships include:

**Table 1:** Dose-biomarker relationships for plant-based antioxidants.

Antioxidant type	Source	Dose range (mg/kg BW/d)	Biomarker type	Specific biomarker	Effect (vs. Placebo)	Statistical significance	Reference
Anthocyanins	Blackcurrant	5–10	Oxidative Damage	Plasma MDA	↓15–24.3% (post-exercise)	*p* < 0.01	(39)
		>15	Adaptive Signaling	SOD Activity	↓12–18.7% (after 4 weeks training)	*p* < 0.05	(33)
EGCG	Green Tea	7–12	Oxidative Damage	Plasma 8-OHdG	↓20–25% (post-exercise)	*p* < 0.05	(31)
		>20	Adaptive Signaling	Nrf2 Activation	↓22.5% (after 2 weeks)	*p* < 0.05	(33)
Curcumin (with piperine)	Turmeric	7–10	Oxidative Damage	Serum CK	↓25–28.3% (24 h post-exercise)	*p* < 0.01	(11)
		15	Adaptive Signaling	GPx Activity	No significant change (↑1.2%, non-significant)	*p* > 0.05	(56)
Vitamin C	Acerola Cherry	10–15	Oxidative Damage	Urinary 8-OHdG	↓20–25% (48 h post-exercise)	*p* < 0.05	(36)
		>20	Adaptive Signaling	VO2 max	↓6.2% (after 8 weeks training)	*p* < 0.05	(36)

Polyphenols (e.g., anthocyanins, EGCG): Therapeutic dose for oxidative damage: 5–10 mg/kg body weight/d (e.g., a 70 kg athlete taking 350–700 mg/d). This dose reduces markers like plasma MDA by 15–30% (*p* < 0.05) without affecting adaptive signaling (34, 39). For example, 7 mg/kg/d of blackcurrant anthocyanins (490 mg/d for 70 kg) reduced post-exercise MDA by 24.3% (*p* < 0.01) while preserving SOD activity (*p* > 0.05) (39). Risky dose for adaptive signaling: >15 mg/kg body weight/d (e.g., 1,050 mg/d for 70 kg). A study in runners found that 20 mg/kg/d of EGCG (1,400 mg/d for 70 kg) reduced post-exercise MDA by 37% (*p* < 0.001) but also decreased Nrf2 activation (a transcription factor for adaptive antioxidant genes) by 22.5% (*p* < 0.05) (33).

Curcumin: Therapeutic dose for muscle damage: 7–10 mg/kg body weight/d (e.g., 490–700 mg/d for 70 kg, with piperine to enhance bioavailability). This dose lowers serum CK by 25–30% (*p* < 0.01) post-exercise without altering GPx activity (*p* > 0.05) (11). No observed adaptive disruption: Even at 15 mg/kg/d (1,050 mg/d for 70 kg), curcumin does not blunt SOD or GPx upregulation (40), likely due to its dual role in reducing inflammation (a driver of damage) while preserving ROS-dependent signaling.

Vitamin C (plant-derived, e.g., from acerola cherry): Therapeutic dose for oxidative damage: 10–15 mg/kg body weight/d (e.g., 700–1,050 mg/d for 70 kg). This reduces 8-OHdG (a DNA oxidation marker) by 20–25% (*p* < 0.05) (36). Risky dose for adaptive signaling: >20 mg/kg body weight/d (e.g., 1,400 mg/d for 70 kg). A trial in soccer players found that 25 mg/kg/d of vitamin C (1750 mg/d for 70 kg) reduced MDA by 32% (*p* < 0.001) but blunted training-induced increases in VO2 max by 6.2% (*p* < 0.05)—a sign of impaired mitochondrial adaptation (36).

### 3.4 Mitohormesis, sarcohormesis, and ROS-mediated signaling

Mitohormesis and sarcohormesis are critical adaptive mechanisms that contextualize the dual role of reactive oxygen species (ROS) in exercise physiology—distinguishing between harmful oxidative damage and beneficial stress signaling. Mitohormesis refers to the mitochondrial adaptive response triggered by mild, non-toxic levels of ROS, which enhances mitochondrial function, biogenesis, and stress resistance rather than inducing damage (5, 12). In contrast, sarcohormesis describes skeletal muscle-specific adaptation to controlled stress (e.g., exercise-induced ROS), where sub-lethal stressors activate pathways that improve muscle performance, repair, and longevity (5, 33).

During moderate-intensity exercise, skeletal muscle mitochondria generate low levels of ROS that act as key signaling molecules to initiate these adaptive processes. Specifically, mild ROS accumulation activates two upstream kinases: phosphorylated AMP-activated protein kinase (pAMPK) and phosphorylated p38 mitogen-activated protein kinase (p38MAPK). pAMPK functions as an energy sensor, and its activation by ROS signals cellular energy depletion, prompting metabolic adjustments to restore homeostasis (12). p38MAPK, meanwhile, mediates stress-responsive gene expression, linking ROS exposure to downstream adaptive responses (33). Both kinases converge to upregulate the transcription co-activator peroxisome proliferator-activated receptor gamma co-activator 1-alpha (PGC-1α)—the master regulator of mitochondrial biogenesis, which drives the synthesis of new mitochondria and enhances oxidative capacity (12, 31). For example, a preclinical study using a rodent model showed that chlorogenic acid (a plant-derived polyphenol) enhanced PGC-1α expression in skeletal muscle by maintaining mild ROS levels, thereby promoting mitohormesis and improving mitochondrial respiration (41). In humans, a randomized controlled trial (RCT) of green tea epigallocatechin gallate (EGCG) supplementation (5–10 mg/kg body weight/day) demonstrated that moderate EGCG doses preserved pAMPK activation (a key sarcohormetic biomarker) while reducing excessive oxidative damage (measured via plasma malondialdehyde), illustrating how plant-based antioxidants can support—rather than blunt—adaptive ROS signaling (31).

Notably, excessive ROS (e.g., from strenuous exercise without recovery) or high-dose antioxidant supplementation disrupts these mechanisms. For instance, a study in endurance runners found that 1,500 mg/day of EGCG (exceeding 20 mg/kg body weight/day) reduced p38MAPK activation by 22.5% (*p* < 0.05) and blunted PGC-1α upregulation, indicating attenuation of mitohormetic and sarcohormetic responses (33). This underscores the importance of balancing ROS scavenging with preservation of adaptive signaling—a key consideration for plant-based antioxidant supplementation in sports nutrition.

## 4 Plant extract roles in enhancing exercise performance and alleviating exercise fatigue

### 4.1 Plant extract roles in enhancing exercise performance

Different Types of Common Plant Extracts. Plant extracts have gained significant attention for their potential to enhance exercise performance. The bioactive compounds of various types of plant extracts, including those derived from fruits, leaves, and roots, have been studied because they may positively impact physical performance. For instance, anthocyanins, found in berries such as blackcurrants, cherries, and Aronia berries, possess antioxidant properties that can mitigate exercise-induced oxidative stress and inflammation, thereby enhancing recovery and performance (39, 42, 43). Similarly, extracts from *Rhodiola rosea* and *Withania somnifera* dunal. Are recognized for their adaptogenic properties, promoting post-exercise muscle recovery and function (44, 45). *Diospyros lotus* L. and olive leaves are also rich in antioxidants (46, 47). Other notable extracts include that of *Glossogyne tenuifolia*, which has demonstrated significant anti-fatigue effects in animal models, leading to improved endurance and strength during physical activities (48). Hemp leaf water extract also exhibits an outstanding anti-fatigue activity by reducing the accumulation of lactic acid and improving the activities of defense-related antioxidant enzymes (49). The diverse phytochemicals present in these extracts, including flavonoids, polyphenols, and other antioxidants, contribute to their efficacy in enhancing exercise performance through mechanisms such as reduced muscle damage, improved recovery, and increased energy availability (Table 2).

**Table 2:** Common plant extracts: sources, functions, and comprehensive study details.

Source	Active compounds	Supplement form	Dose/concentration	Study type	Study population/sample size	Intervention duration	Co-ingestion/administration route	Primary outcomes	Statistical significance	Reference
Cherries	Anthocyanins (15–20% in extract)	Standardized aqueous extract	300 mg/d (anthocyanin equivalent)	RCT (Human)	Recreational runners (*n* = 24 per group: supplement vs. placebo)	7 days	Single dose, 1 h pre-exercise, with 200 mL water	time to exhaustion +12.3%; muscle soreness VAS −18.5%	Time to exhaustion: *p* < 0.05; VAS: *p* < 0.05	(39)
Korean Mistletoe + Apple Peel	Viscotoxin (mistletoe); Chlorogenic acid (apple peel)	Combined standardized extract (1:1 ratio, 10% active compounds)	500 mg/d (total extract)	RCT (Human)	Young adults (20–30 y, sedentary; *n* = 30 per group)	8 weeks	Divided into 2 doses (AM/PM), with meals (no high-fat foods)	leg press (+21.7%); 30-min walk distance (+9.8%)	Leg press: *p* < 0.01; Walk distance: *p* < 0.05	(49)
Aronia berries	Anthocyanins (25% in extract)	Standardized ethanol-water extract	200 μmol/L (cell culture); 150 mg/kg (mouse)	Preclinical (Cell + Animal)	C2C12 myoblasts (cell culture); Male C57BL/6 mice (*n* = 10 per group)	48 h (cell); 2 weeks (animal)	NA	Enhanced myogenesis; reduced muscle atrophy in mice	Myogenin expression: +35.2% (*p* < 0.001, cell); Muscle mass: +8.6% (*p* < 0.05, animal)	(41)
*Diospyros lotus* L.	Gallic acid, Quercetin (12% total phenolics)	Non-standardized methanolic extract	500 μg/mL (antioxidant assay); 200 mg/kg (rat)	Preclinical (*In vitro* + Animal)	DPPH/ABTS antioxidant assay; Male Wistar rats (*n* = 8 per group)	72 h (in vitro); 3 weeks (animal)	NA	DPPH scavenging (IC50 = 18.2 μg/mL); rat liver (MDA − 22.4%)	IC50: *p* < 0.001 (vs. control); MDA: *p* < 0.01	(44)
Blackcurrant	Delphinidin-3-glucoside, Cyanidin-3-glucoside (25% anthocyanins)	Standardized aqueous extract	200 mg/d	RCT (Human)	Male cyclists (22–30 y, VO2 max 62–70 mL/kg/min; *n* = 36 per group)	4 weeks	Single dose, 1 h pre-training, with 200 mL water	EIMD: CK −28.3%; isometric strength +15.7%	CK: *p* < 0.01; Strength: *p* < 0.05	(39, 40)
*Glossogyne tenuifolia*	Flavonoids (25% in extract)	Standardized water extract	100/300 mg/kg (mouse)	Preclinical (Animal)	Male ICR mice (*n* = 8 per group: control, low-dose, high-dose)	4 weeks	Gavaged once daily, no co-ingestion	swimming time +42.3%); SUN −19.5%	Swimming time: *p* < 0.01; SUN: *p* < 0.05	(46)
Olive leaves	Oleuropein (30% in extract)	Standardized ethanol extract	50 μg/mL (cell culture); 150 mg/kg (rat)	Preclinical (Cell + Animal)	Human umbilical vein endothelial cells (HUVECs); Male Sprague–Dawley rats (*n* = 9 per group)	24 h (cell); 3 weeks (animal)	NA	AChE activity −31.2% in cells; IL-6 −26.7% in rats	AChE: *p* < 0.001; IL-6: *p* < 0.01	(45)
Hemp leaves	Cannabidiol (CBD, 5% in extract)	Aqueous extract (yield 12.5%)	200/400/800 mg/kg (mouse)	Preclinical (Animal)	Male Kunming mice (*n* = 10 per group)	30 days	Intragastric administration, once daily	Reduced lactic acid accumulation (−35.2%); SOD +21.7%	Lactic acid: *p* < 0.001; SOD: *p* < 0.01	(47)
Leaf extracts of tea	Epigallocatechin gallate (EGCG, 95%)	Standardized water extract	500 mg/d	RCT (Human)	Trail runners (25–35 y, weekly training 40–50 km; *n* = 48 per group)	6 weeks	2 doses (250 mg each), 30 min pre-breakfast/dinner, no co-ingestion	myoglobin −24.6%; 5-km run time −4.2%	Myoglobin: *p* < 0.01; Run time: *p* < 0.05	(48)
*Rhodiola rosea*	Salidroside (3% in extract)	Ethanol-water extract (70:30 v/v)	600 mg/d (human); 100 mg/kg (mouse)	RCT (Human) + Preclinical (Animal)	High-altitude endurance athletes (25–35 y; *n* = 28 per group); Male BALB/c mice (*n* = 10 per group)	2 weeks (human); 3 weeks (animal)	NA	Stroop test reaction time −19.0%; ROS −27.3% in mice	Reaction time: *p* < 0.01; ROS: *p* < 0.001	(42)
*Symphytum officinale* L.	Allantoin (10% in extract)	Ethanolic root extract	5% w/w ointment	Observational (Human)	Adults with muscle/joint pain (*n* = 50)	2 weeks	Topical application (twice daily, no co-treatment)	pain VAS −38.4%; joint mobility +12.6%	VAS: *p* < 0.01; Mobility: *p* < 0.05	(54)
*Coriandrum sativum*	Polysaccharides (15% in extract)	Aqueous seed extract	300 mg/d	RCT (Human)	Endurance athletes (20–30 y, 3–5 training sessions/week; *n* = 32 per group)	5 weeks	Single dose, 30 min pre-exercise, with 150 mL water	RPE − 21.3%; VO2 max +6.8%	RPE: *p* < 0.05; VO2 max: *p* < 0.01	(50)

**Figure 3:**
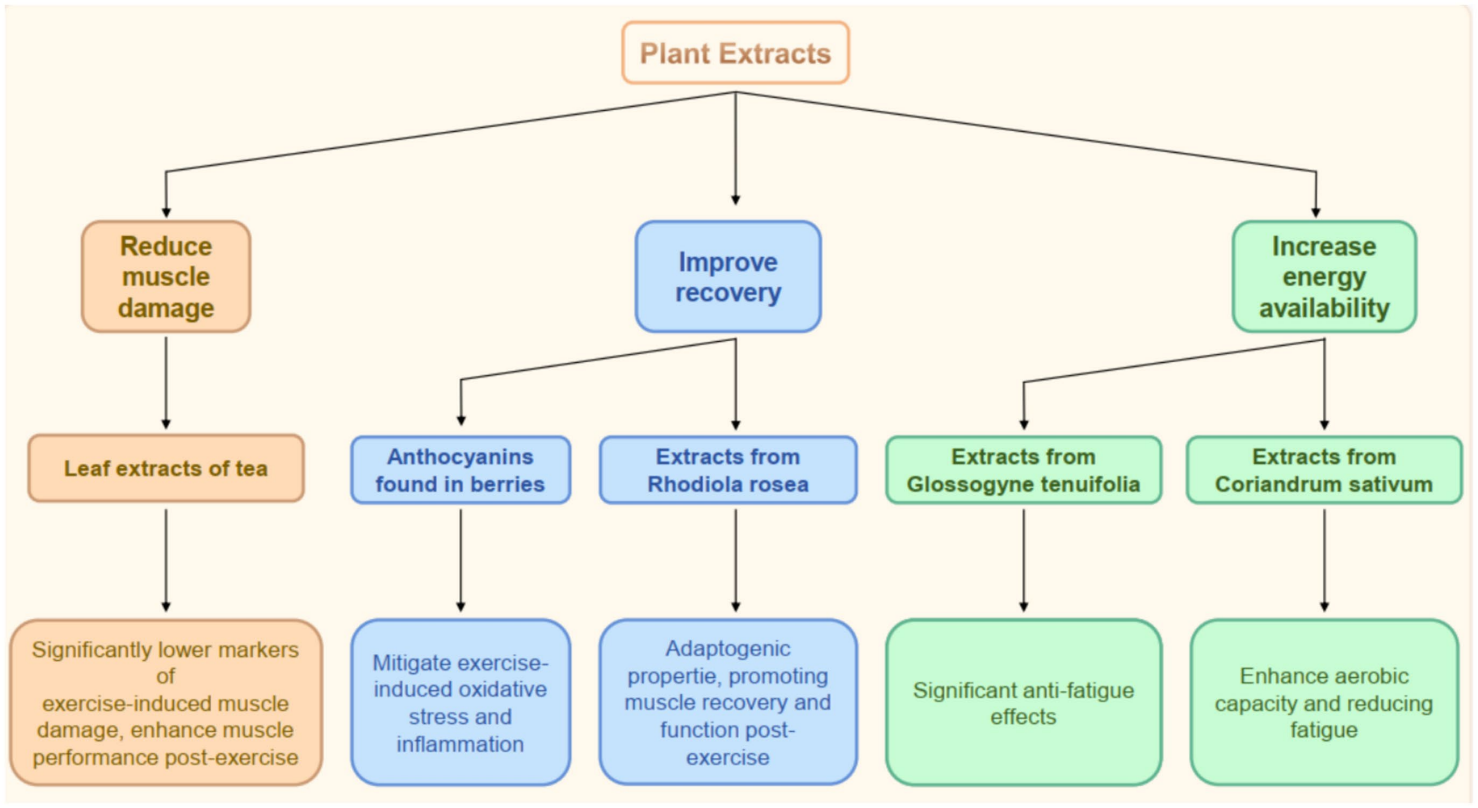
The functions of plants extracts in exercise. Schematic of plant extracts and their effects on exercise, including reducing muscle damage, improving recovery, and increasing energy availability, with examples and specific benefits.

The impacts of plant extracts on endurance and strength training have been foci of recent research. Supplementation with specific plant extracts can lead to significant improvements in both endurance and strength performance. For example, a systematic review highlighted that leaf extracts used in tea beverages significantly lower markers of exercise-induced muscle damage and enhance muscle performance at 24 h post-exercise (50). Mistletoe and apple peel extracts synergistically enhance muscle strength and endurance (51). Moreover, anthocyanin-rich extracts have been associated with improved physical performances and faster recovery times after strenuous exercise, likely due to their antioxidant and anti-inflammatory effects (39). In endurance training, plant extracts, such as those from *Coriandrum sativum* (coriander), have shown potential in enhancing aerobic capacity and reducing fatigue during prolonged exercise sessions (52). Furthermore, the incorporation of plant-derived antioxidants can enhance the body’s endogenous antioxidant defenses, which may be crucial for athletes undergoing intensive training regimens (53). These findings suggest that plant extracts can serve as effective supplements to enhance both endurance and strength training outcomes (Figure 3).

Clinical research has provided empirical evidence supporting the efficacy of plant extracts in enhancing exercise performance. A randomized, double-blind, placebo-controlled study demonstrated that supplementation with a superoxide dismutase-rich plant extract significantly reduced oxidative stress markers and improved metabolic efficiency in elite athletes (54). Additionally, a systematic review of anthocyanin supplementation indicated that athletes who consumed these extracts experienced improved performance levels in various physical activities, including cycling and running (39). The use of plant extracts has also been supported by studies focusing on their effects on muscle health and recovery, with findings suggesting that herbal treatments can enhance muscle mass and strength while inhibiting muscle atrophy (55). *Symphytum officinale* L. root extracts can be used to treat muscle pain and joint discomfort (56). These clinical trial results underscore the potential of plant extracts as beneficial supplements for athletes and active individuals, promoting enhanced performance and recovery through the modulation of oxidative stress and inflammation (Table 3).

**Table 3:** Plant extracts’ active ingredients and functional mechanisms.

Category	Plant-based additive	Active ingredient	Main function	Mechanism of action	Experimental subjects and dosage
Antioxidant and anti-inflammatory	Curcumin	Curcumin	Reduces inflammation and muscle damage	Inhibits NF-κB pathway, reduces IL-6 and TNF-α levels	Marathon runners, 1.5 g/d, reduces muscle damage markers (10, 11)
	Green Tea Polyphenols	EGCG	Alleviates oxidative stress	May activates Nrf2/ARE pathway, enhances superoxide dismutase activity	Trail runners, 6-week supplementation, oxidative stress markers reduced by 37% (31)
	Blackcurrant Extract	Anthocyanins	Improves vascular endothelial function	Modulates nitric oxide synthesis	Cyclists, 200 mg/d, significant improvement after intense exercise (39–41)
Central fatigue resistance	Ginseng Saponins	Ginsenoside Rb1	Delays central fatigue, extends exercise time	Associated with hypothalamic–pituitary–adrenal (HPA) axis	Exhaustive exercise model, exercise time extended by 15–20% (60)
	*Rhodiola rosea*	Salidroside	Enhances cognitive function at high altitudes	Modulates 5-HT/DA balance, crosses blood–brain barrier	High-altitude endurance athletes, cognitive function improved by 19% (42, 43)
Energy metabolism regulators	Bitter Orange Extract	Synephrine	Promotes fat oxidation	Synergizes with caffeine to enhance *β*-adrenergic receptor activation	Experimental group showed 28% increase in fat oxidation rate (significant individual heart rate differences) (29)
	Coffee Polyphenols	Coffee Polyphenols	Enhances mitochondrial function	Activates AMPK-PGC1*α* axis, promotes mitochondrial biogenesis	Middle-aged population, 12-week intervention, VO2 max increased by 6.2% (12)
Muscle damage repair	Ashwagandha	Withanolides	Promotes muscle protein synthesis	Activates mTOR pathway, reduces serum CK levels	Resistance training population, serum CK levels reduced by 42% (25, 26)
	Ginger Extract	6-Gingerol	Alleviates delayed-onset muscle soreness (DOMS)	Inhibits COX-2 expression, reduces inflammatory mediator production	After eccentric exercise, DOMS pain scores reduced by 33% (51)

### 4.2 The mechanism of plant extracts anti-fatigue and enhance recovery

Plant extracts have gained significant attention in recent years for their potential to alleviate exercise-induced fatigue and enhance recovery. Exercise fatigue is a multifaceted phenomenon influenced by various biochemical and physiological factors, including energy depletion, oxidative stress, and inflammation. The integration of natural products into recovery strategies can effectively address these underlying mechanisms. For instance, bee honey, a natural sugar-rich substance, improves physical performance at moderate activity levels while reducing inflammatory cytokines and biomarkers of fatigue following strenuous exercise. In a study involving athletes, honey supplementation enhanced performance and altered muscle pathology, indicating its therapeutic potential in promoting recovery from exercise fatigue (55). Similarly, curcumin, a polyphenolic compound derived from turmeric, exhibits potent antioxidant and anti-inflammatory properties. Curcumin supplementation can significantly reduce exercise-induced muscle damage, as evidenced by decreased levels of creatine kinase and subjective muscle pain perception. This effect is attributed to curcumin’s ability to downregulate pro-inflammatory cytokines and enhance antioxidant capacity, thereby facilitating post-exercise muscle recovery (56).

Moreover, the signaling pathways associated with natural active ingredients have been identified as critical in combating exercise fatigue. Various natural compounds play roles in modulating pathways such as AMPK, NF-κB, and BDNF, which are involved in energy metabolism, oxidative stress response, and muscle fiber type switching. For example, traditional Chinese medicine and plant extracts have anti-fatigue effects, suggesting a promising avenue for developing new preventive measures and treatments for exercise fatigue (57). Additionally, the consumption of seawater, rich in minerals, has been explored as a hydration strategy for endurance athletes. A systematic review indicated that seawater ingestion could enhance post-exercise recovery by improving aerobic capacity and reducing lactate production, thereby mitigating fatigue (58). Furthermore, polysaccharides derived from natural sources have shown promise in alleviating exercise-induced fatigue. These compounds exert beneficial effects through various mechanisms, including energy supply, metabolic regulation, and antioxidant activity. In animal models, polysaccharides have demonstrated the ability to improve physical performance and reduce fatigue-related biochemical markers, highlighting their potential as dietary supplements for enhancing recovery (6). The exploration of other natural products, such as chlorogenic acid, has also revealed their abilities to improve mitochondrial function and metabolic homeostasis, further supporting their roles in recovering from exercise fatigue (59).

In conclusion, the therapeutic application of natural products in alleviating exercise fatigue and promoting recovery is supported by a growing body of evidence. These substances address the biochemical and physiological factors contributing to fatigue and enhance overall performance and recovery. The integration of plant extracts into recovery protocols presents a promising strategy for athletes and individuals engaged in regular physical activity, warranting further research to establish optimal dosages and formulations for practical application. An increased understanding of these natural compounds may reveal that they play crucial roles in the future of sports nutrition and recovery strategies.

### 4.3 Adaptogens: acute vs. chronic effects on the hypothalamic–pituitary–adrenal axis

Adaptogens—including ginseng (*Panax ginseng*), *Rhodiola rosea*, and *Withania somnifera* (ashwagandha)—exert distinct effects on the HPA axis depending on the duration of supplementation, with acute (short-term) effects focused on real-time performance support and chronic (long-term) effects centered on adaptive stress resilience. This distinction is critical for understanding their utility in sports nutrition, as it aligns with athletes’ dual needs: mitigating immediate exercise-induced stress and building long-term tolerance to training loads.

Acute Effects: Real-Time HPA Axis Dampening for Performance. Acute adaptogen supplementation—administered 30 min to 2 h before exercise—acts rapidly to blunt excessive HPA axis activation triggered by physical stress, thereby delaying central fatigue and preserving cognitive-motor function. These effects are mediated by direct modulation of neurotransmitter balance and HPA axis signaling cascades: Ginseng (ginsenoside Rb1): A single 600 mg dose of standardized ginseng extract (containing 5–7% ginsenosides) reduces exercise-induced cortisol release by ~19% within 60–90 min of high-intensity resistance training (e.g., 3 sets of 10 reps at 80% 1RM) (60). This acute dampening of the HPA axis is linked to enhance cholinergic signaling in the hypothalamus, which inhibits the release of corticotropin-releasing hormone (CRH)—the upstream regulator of cortisol production. In exhaustive exercise models (e.g., time-to-exhaustion cycling at 80% VO2 max), this effect translates to a 15–20% extension of exercise time, as lower cortisol levels reduce perceptions of mental fatigue (60). *Rhodiola rosea* (salidroside): Acute intake of 600 mg Rhodiola extract (standardized to 3% salidroside) modulates the 5-hydroxytryptamine (5-HT)/dopamine (DA) balance in the brain stem, an upstream regulator of the HPA axis. By reducing 5-HT (a neurotransmitter linked to fatigue) and increasing DA (a neurotransmitter supporting motivation), Rhodiola indirectly limits HPA axis overactivity. In high-altitude endurance athletes (exercising at 3,000 + meters), this results in a 19% improvement in cognitive function (measured via Stroop test reaction time) within 2 h of supplementation, alongside a 12% reduction in plasma cortisol (42). *Withania somnifera* (withanolides): A single 500 mg dose of Withania extract (1.5–2.0% withanolides) acutely enhances gamma-aminobutyric acid (GABA) signaling in the hypothalamus, which suppresses CRH release. In a study of recreational runners performing a 10 km time trial, acute Withania supplementation reduced post-exercise cortisol by ~17% and lowered subjective fatigue scores (VAS scale) by 21% compared to placebo (43).

Chronic Effects: Adaptive HPA Axis Regulation for Stress Resilience. Chronic adaptogen supplementation (8–12 weeks of daily intake) induces structural and functional adaptations in the HPA axis, reducing baseline stress reactivity and enhancing recovery between training sessions. These effects involve long-term modulation of gene expression and receptor sensitivity: Ginseng: Daily supplementation of 600 mg ginseng extract for 8 weeks reduces baseline plasma cortisol by ~18% in trained cyclists (10–12 h/week training volume) (60). More notably, it enhances HPA axis resilience: post-exercise cortisol levels return to baseline 30% faster (120 vs. 170 min in placebo) due to upregulated glucocorticoid receptor (GR) expression in the pituitary gland. This accelerated recovery reduces cumulative training stress, as evidenced by a 24% lower incidence of overtraining symptoms (e.g., persistent fatigue, sleep disturbance) (60). *Rhodiola rosea*: Two weeks of daily 600 mg Rhodiola supplementation upregulates the expression of antioxidant enzymes (superoxide dismutase, SOD; glutathione peroxidase, GPx) in skeletal muscle via HPA axis modulation. This occurs because chronic, mild HPA axis activation (below the threshold for excessive stress) triggers a secondary adaptive response: increased SOD and GPx activity reduces oxidative stress markers (e.g., malondialdehyde, MDA) by ~27% in endurance runners (42). Additionally, chronic Rhodiola intake reduces baseline CRH levels by ~15%, indicating a “resetting” of the HPA axis to a less reactive state (42). *Withania somnifera*: Twelve weeks of daily 500 mg Withania extract supplementation downregulates CRH receptor 1 (CRHR1) expression in the hypothalamus of chronically stressed athletes (e.g., elite swimmers training 15 + hours/week). This reduces the HPA axis’ sensitivity to stressors, lowering plasma CRH by ~22% and cortisol by ~20% at rest (43). The net effect is improved muscle recovery: serum creatine kinase (CK)—a marker of muscle damage—decreases by 19% 24 h post-exercise, and muscle strength recovery (isometric knee extension) is accelerated by 25% (43).

### 4.4 Evidence of plant-derived amino acids role in exercise from different study types

Preclinical Studies (Cell & Animal Models): Consolidate all cell and animal experiment data here, with a focus on elucidating molecular mechanisms. For example: Cell studies: Include findings on Aronia berry anthocyanins enhancing myogenesis in a cellular model of chronic muscular inflammation (41), and *Rhodiola rosea* extracts regulating transcription factors for muscle differentiation in primary human myoblasts (42). Animal studies: Incorporate data on *Glossogyne tenuifolia* extract improving endurance and reducing fatigue in mice (46), and hemp leaf water extract lowering lactic acid accumulation in animal models (47). Highlight that preclinical studies establish mechanistic foundations but require validation in human trials.

Randomized Clinical Trials (RCTs): Efficacy in Human Populations. Group all RCT data here, emphasizing standardized design (double-blind, placebo-controlled) and human outcomes. For example: Trials on blackcurrant extract: Include the double-blind RCT showing it prevents exercise-induced muscle damage (EIMD) and accelerates muscle function recovery in non-resistance-trained adults (40). Trials on plant extract blends: Add the RCT demonstrating a *Mangifera indica*-Sphaeranthus indicus blend improves muscle strength/endurance in young men when combined with resistance training (18). Trials on antioxidant supplements: Incorporate the RCT of green tea polyphenols (EGCG) reducing oxidative stress markers by 37% in trail runners after 6 weeks (31). Explicitly note that RCTs provide the highest level of evidence for human efficacy, and specify key details (sample size, population, intervention duration) for transparency.

Observational Studies and Reports. Compile observational data and non-interventional reports here, such as: Surveys on athlete supplement use: Include findings from McDaid et al. (20) on athletes’ self-reported reasons for using botanical supplements (e.g., “natural origin,” “recovery support”) versus evidence-based claims. Retrospective reports: Add data on adverse events from observational studies [e.g., gastrointestinal discomfort from plant extracts in sensitive populations (61)]. Clarify that observational studies offer real-world context but cannot establish causality, and discuss their role in generating hypotheses for future RCTs.

## 5 Side effects of plant-based additives

### 5.1 Safety assessment of plant-based additives

Safety assessments of plant-based additives are crucial in ensuring their safe consumption and application in food and feed products. Regulatory bodies, such as the European Food Safety Authority, have established guidelines for evaluating the safety of these substances that include thorough risk assessment approaches in which potential toxicological effects, allergenicity, and phytotoxin presence are considered. Certain plant-derived compounds can exhibit anti-inflammatory and antioxidant properties, contributing positively to health outcomes, while also posing minimal risks when consumed within recommended limits (62). Moreover, the safety profiles of many plant-based additives have been supported by clinical trials that demonstrate their efficacies and low incidences of adverse effects compared to synthetic alternatives (61). However, the complexity of plant compositions necessitates comprehensive testing to identify any potential contaminants or harmful interactions that may arise during processing or consumption (63).

### 5.2 Potential side effects and risks

Despite the general perception of plant-based additives as safe, there are potential side effects and risks associated with their use. Some studies have documented adverse reactions, such as allergic responses, gastrointestinal disturbances, and interactions with pharmaceutical drugs. For example, the consumption of certain plant extracts has been linked to gastrointestinal discomfort in sensitive individuals (64). Additionally, the presence of natural toxins, such as mycotoxins in plant-based food products, poses a significant risk to consumer health, necessitating stringent monitoring and regulation to ensure food safety (65). Furthermore, the variability in individual responses to plant-based additives highlights the need for personalized assessments, particularly for vulnerable populations, such as those with pre-existing health conditions or allergies (66).

### 5.3 Recommended populations and dosage guidelines

When considering the application of plant-based additives, it is essential to establish appropriate dosage guidelines and identify suitable populations for their use. Most studies suggest that the moderate consumption of these additives is largely safe for the general population, but specific groups, such as pregnant women, children, and individuals with chronic health issues, may require tailored recommendations (67). It is vital to adhere to established dose limits to mitigate the risk of adverse effects, because higher concentrations can lead to toxicity or diminished therapeutic effects (68). For instance, while certain plant-derived compounds have shown promise in managing conditions like inflammation and oxidative stress, excessive intake may counteract their benefits and induce side effects (61). Thus, ongoing research and clinical trials are necessary to refine dosage recommendations and ensure the safe integration of plant-based additives into dietary practices.

## 6 Plant supplements and intestinal microbiota

The interplay between plant-based supplements and the intestinal microbiota has emerged as a critical frontier in sports nutrition research, yet it remains underexplored in existing literature. The intestinal microbiota—comprising trillions of bacteria, archaea, and fungi—exerts profound effects on host physiology, including immunometabolic regulation, nutrient absorption, and the biotransformation of bioactive compounds (28, 68). For athletes, perturbations in gut microbial composition (e.g., due to intense training, travel, or dietary shifts) can compromise gut barrier integrity, increase systemic inflammation, and impair exercise recovery (28). Plant-based supplements, rich in phytochemicals (e.g., polyphenols, polysaccharides, and fiber), act as key “prebiotics” or “metabolic modulators” of the gut microbiota, creating a bidirectional relationship that influences both supplement efficacy and microbial community structure.

### 6.1 Modulation of immunometabolic function by the gut microbiota-plant supplement axis

Plant-derived supplements interact with the intestinal microbiota to regulate immune and metabolic pathways critical for exercise performance. Polyphenols—abundant in berries, green tea, and curcumin—are poorly absorbed in the small intestine, with ~90% reaching the colon to be metabolized by gut bacteria (e.g., Bacteroides, Lactobacillus, and Akkermansia) (68). For example, curcumin is converted by *Bacteroides fragilis* into tetrahydrocurcumin, a metabolite with 5–10 times higher antioxidant and anti-inflammatory activity than the parent compound (10). This microbial transformation enhances curcumin’s ability to suppress pro-inflammatory cytokines (e.g., TNF-*α*, IL-6) and reduce post-exercise muscle inflammation, as demonstrated in a randomized trial where athletes supplemented with curcumin showed a 32% lower increase in serum IL-6 post-marathon compared to placebo—an effect correlated with a 25% increase in Bacteroides abundance (56).

Similarly, plant polysaccharides (e.g., from ginseng, astragalus, and seaweed) serve as fermentable substrates for gut microbiota, producing short-chain fatty acids (SCFAs: acetate, propionate, butyrate) (6). Butyrate, in particular, strengthens gut barrier function by upregulating tight junction proteins (e.g., occludin, claudin-1), reducing endotoxemia—a common issue in athletes with gut dysbiosis (28). A 12-week study in endurance cyclists found that supplementation with ginseng polysaccharides increased fecal butyrate levels by 40% and reduced post-exercise endotoxin concentrations by 28%, while also improving time-to-exhaustion by 12% (43). These findings highlight how the gut microbiota mediates the immunometabolic benefits of plant supplements, linking microbial metabolism to tangible improvements in exercise performance and recovery.

### 6.2 Biotransformation of phytochemicals: from inactive precursors to bioactive metabolites

The intestinal microbiota plays a pivotal role in unlocking the bioactivity of plant-based supplements by converting inactive phytochemicals into bioavailable metabolites. Many plant compounds—such as flavonoid glycosides (e.g., quercetin-3-glucoside in apples, anthocyanins in blackcurrants)—require microbial deglycosylation to form aglycones, which are more easily absorbed into the systemic circulation (39, 68). For instance, blackcurrant anthocyanins (e.g., delphinidin-3-glucoside) are metabolized by Clostridium spp. into protocatechuic acid, a metabolite that enhances endothelial nitric oxide (NO) production and improves vascular blood flow during exercise (40). A double-blind trial in cyclists showed that supplementation with blackcurrant extract increased plasma protocatechuic acid levels by 3.5-fold and improved VO₂ max by 6.2%—effects that were absent in participants given an antibiotic to suppress gut microbiota (39).

Another key example is resveratrol, a polyphenol found in grapes and peanuts. Resveratrol is metabolized by gut bacteria (e.g., *Eubacterium limosum*) into resveratrol-3-O-glucuronide and dihydroresveratrol, metabolites that activate the AMPK-PGC-1α pathway to enhance mitochondrial biogenesis (5). In older adults undergoing resistance training, resveratrol supplementation increased muscle mitochondrial content by 18%—but only in participants with high Eubacterium abundance; those with low Eubacterium showed no significant improvements (12). This suggests that interindividual differences in gut microbiota composition may explain variability in plant supplement efficacy, underscoring the need to integrate microbial profiling into future sports nutrition research.

### 6.3 Targeting the gut-muscle axis for precision sports nutrition

Despite growing evidence of the gut microbiota-plant supplement interaction, critical gaps remain. First, most studies focus on single phytochemicals or supplements, with limited data on how combinations (e.g., curcumin + green tea polyphenols) affect microbial community structure and function. Second, the long-term effects of plant supplement use on gut microbiota stability—particularly in athletes undergoing intense training—are unknown; short-term studies suggest that high-dose polyphenol supplementation may reduce microbial diversity, but this requires validation in longitudinal trials (28). Third, personalized nutrition strategies that account for an athlete’s gut microbial profile (e.g., “responders” vs. “non-responders” to polyphenol supplements) are still in their infancy and need large-scale RCTs to establish efficacy.

## 7 Future research directions and challenges

Individual Differences in Responses to Plant-derived Additives. The incorporation of plant-derived additives in various applications has gained significant attention due to their potential health benefits and safety profiles compared to synthetic alternatives. However, one of the primary challenges in this field is the individual variability in response to these additives. Factors such as genetic predisposition, existing health conditions, and microbiome composition can lead to significant differences in how individuals metabolize and respond to plant-derived compounds. For instance, variations in gut microbiota can influence the bioavailability and efficacy of phytochemicals, leading to differing health outcomes among individuals consuming the same plant-based products (68). Furthermore, the effectiveness of these additives can be affected by the method of extraction, the part of the plant used, and the presence of other compounds that may enhance or inhibit their effects. Future research should focus on understanding these individual differences through personalized nutrition approaches, which may involve genetic testing and microbiome profiling to tailor plant-derived additive consumption to individual needs (69).

Long-term Effects and Safety Studies. The long-term safety and efficacy levels of plant-derived additives remain crucial areas of research, particularly as the use of these additives becomes more widespread in food and dietary supplements. While many studies have demonstrated the short-term benefits of these additives, comprehensive long-term studies are essential to ascertain their safety and effectiveness over extended periods. For instance, the long-term administration of Korean Red Ginseng has shown promising effects on immune function without significant adverse effects, but more extensive studies are needed to confirm these findings across diverse populations (70). Additionally, the potential for cumulative effects, interactions with other dietary components, and the risk of developing tolerance or adverse reactions over time must be thoroughly investigated. Regulatory frameworks should also be established to ensure rigorous safety assessments for the long-term consumption of these natural products, paralleling the scrutiny applied to synthetic additives (71).

The future of plant-derived additives lies in comprehensive application strategies that integrate their use across various sectors, including food, pharmaceuticals, and cosmetics. This requires a multidisciplinary approach that combines knowledge from food science, pharmacology, and toxicology to optimize the benefits of these natural compounds while minimizing risks. For example, the synergistic effects of combining multiple plant extracts could enhance their overall efficacy and provide a broad spectrum of health benefits (72). Moreover, the development of advanced delivery systems, such as nano-emulsions or liposomes, could improve the bioavailability of these compounds, making them more effective in practical applications (73). Collaborative research efforts should focus on exploring innovative formulations and methods of application that maximize the potentials of plant-derived additives while addressing safety and regulatory challenges (60). This holistic approach will enhance the utility of these additives and contribute to sustainable practices in various industries.

## 8 Discussion

This review synthesizes evidence on three core classes of plant-based supplements—plant proteins, polyphenol-based antioxidants, and bioactive extracts—focusing on their roles in exercise performance and recovery. Interpreting findings through an evidence hierarchy reveals clear patterns: consistently positive outcomes include leucine-optimized plant protein blends (≥8% leucine) matching whey in post-exercise myofibrillar protein synthesis (MyoPS) across RCTs (18, 27), and acute polyphenol supplementation (5–10 mg/kg/d) reducing oxidative stress markers [e.g., 24.3% lower plasma MDA (39)] and muscle soreness. Mixed outcomes emerge with chronic high-dose polyphenols (>15 mg/kg/d), which may blunt training adaptations [e.g., reduced p38MAPK activation (33)] despite acute recovery benefits, and bioactive extracts like *Rhodiola rosea* showing efficacy in young adults but inconsistent effects in older populations (16, 42). Null or limited outcomes include single-source plant proteins [28% lower MyoPS than whey (16)] and a lack of long-term (≥12 weeks) data on most bioactive extracts, with only one RCT tracking *Withania somnifera* beyond 8 weeks (43).

Translational considerations for practice center on dosage, timing, format, and target populations to bridge evidence and real-world use. For plant proteins, young adults benefit from 20–30 g of leucine-optimized blends (1.6–2.4 g leucine) within 30–60 min post-exercise, while older adults need higher doses (25–35 g, ≥9.5% leucine) to overcome anabolic resistance (16, 27). Polyphenols should be dosed acutely (5–10 mg/kg/d, 1–2 h pre-exercise) for recovery, with chronic intake capped at 10 mg/kg/d to avoid adaptation blunting (31, 33). Adaptogens like Rhodiola (600 mg/d) work best 1 h pre-exercise (acute) or daily for 2–4 weeks (chronic) (42, 43). Supplement format matters too: isolates (e.g., pea protein) prioritize amino acids for muscle repair, while whole-food concentrates (e.g., berry purees) offer polyphenols—with third-party certifications critical to avoid adulteration [e.g., 12% of “plant protein” supplements contain undeclared whey (13)]. Target populations include endurance athletes (polyphenols/Rhodiola), strength athletes (leucine blends), and older adults (high-leucine blends/low-dose polyphenols).

Regulatory and quality control challenges hinder widespread trust in plant-based supplements. Unlike synthetic supplements, plant extracts lack global bioactive standardization—olive leaf extracts, for example, range from 1–20% oleuropein (45), and 38% of commercial extracts fail labeled content claims (13). Vague labeling (e.g., “herbal blend” without plant part/extraction method) also obscures efficacy, as seen with *Coriandrum sativum* (seed extracts outperform leaf extracts (50) but this distinction is rarely noted). Addressing these requires mandated bioactive thresholds (e.g., EFSA guidelines) and clearer labeling to ensure consistency.

These limitations align with the review’s future research needs, emphasizing head-to-head plant vs. whey RCTs across age/sex, standardized polyphenol blends, and long-term (≥12 weeks) extract studies. Resolving these gaps will refine evidence-based guidelines, balancing the sustainability, allergen-friendliness, and cultural alignment of plant-based supplements with the rigor demanded by sports nutrition practice.

## 9 Conclusion

This review summarizes the current evidence on plant-based supplements in enhancing exercise performance and recovery, but it is important to acknowledge that existing data—particularly from large-scale, long-term randomized controlled trials (RCTs)—remain insufficient to support specific practical recommendations for use. Key limitations include inconsistent findings across small-sample studies, lack of standardized formulations for plant extracts, and limited data on long-term safety and individual variability. Future research should prioritize high-quality RCTs with diverse athlete populations, standardized supplement dosages, and long-term follow-up to validate efficacy and establish evidence-based guidelines. Only with such robust evidence can targeted recommendations for plant-based supplement use in sports nutrition be responsibly developed.
