# Supplementary figures and images for: Plant-based supplements in enhancing exercise performance and recovery
Source: Front Nutr. 2025 Nov 24;12:1699642. doi: 10.3389/fnut.2025.1699642 (PMC12682642; doi:10.3389/fnut.2025.1699642)

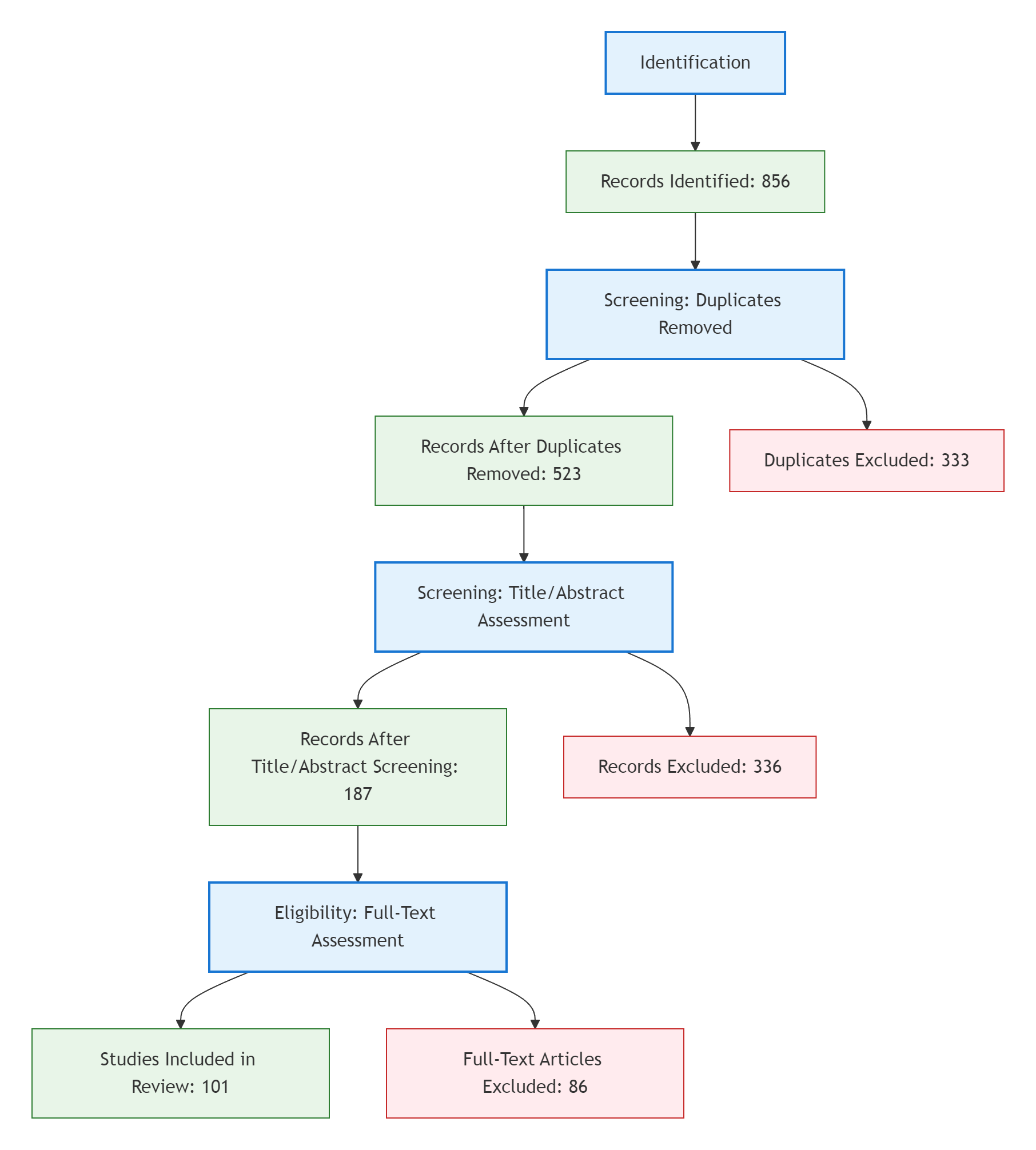

Supplement: Supplementary file 1 [file Image_1.PNG]
